# Supplementary material for: DNA methylation age analysis of rapamycin in common marmosets
Source: GeroScience. 2021 Sep 5;43(5):2413–25. doi: 10.1007/s11357-021-00438-7 (PMC8599537; doi:10.1007/s11357-021-00438-7)
Supplement: Supplementary file 1 — Supplementary file1 (DOCX 90.6 kb) [file 11357_2021_438_MOESM1_ESM.docx]

**SUPPLEMENTARY MATERIAL**

for the article "*DNA methylation age analysis of rapamycin in common marmosets*"

**Supplementary Figure 1**. Unsupervised hierarchical clustering of blood samples from marmosets. Average linkage hierarchical clustering based on the inter-array correlation coefficient (Pearson correlation). The low height values (y-axis) indicate high inter array correlations (R>0.98) and high quality. There is no obvious clustering pattern. The first color band indicates rapamycin treatment status: red=rapamycin treated, black=control, while=not part of the rapamycin study.

**Technical Details surrounding the DNAm age estimator**

**Statistical methods used for building the clocks**

The epigenetic clocks were used by employing a single elastic net regression model analysis (R function glmnet). We use used Leave-one-out analysis (LOO) using a single lambda value. We chose the following parameters for the glmnet R function (Alpha: 0.5, CV Fold: 10, Lambda choice for Clock: 1 standard error above minimum CV-MSE).

**Covariates and coefficient values of the marmoset clocks**

1. The marmoset blood tissue clock is based on 56 CpGs whose coefficient values are specified in the column "Coef.MarmosetPanTissue". Age transformation=identity, i.e. F(Age)=Age
2. The human marmoset clock for chronological age is based on 343 CpGs whose coefficient values are specified in the column "Coef.HumanMarmosetLogLinearAge". Age transformation=log-linear described below.
3. The final human marmoset clock for relative age is based on 385 CpGs whose coefficient values are specified in the column "Coef.HumanMarmosetBloodRelativeAge". Age transformation: relative age. i.e. F(Age)=Age/maxLifespan. Max lifespan for marmosets is

22.8 years. Human max lifespan =122.5 years.

**General description of age transformation**

The human-marmoset clocks for chronological age used log linear transformations that are similar to those employed for the HUMAN pan tissue (Horvath 2013) ^1^.

An elastic net regression model (implemented in the glmnet R function) was used to regress a transformed version of age on the beta values in the training data. The glmnet function requires the user to specify two parameters (alpha and beta). Since I used an elastic net predictor, alpha was set to 0.5. But the lambda value of was chosen by applying a 10 fold cross validation to the training data (via the R function cv.glmnet).

The elastic net regression results in a linear regression model whose coefficients b_0_, b_1_, . . . , relate to transformed age as follows
*F*(chronological age)=*b*_0_*+b*_1_*CpG*_1_*+ . . . +b*_p_*CpG*_p_+error

Note that the intercept term is denoted by b_0_. The coefficient values can be found in the attached Excel file.

Based, on the coefficient values from the regression model, DNAmAge is estimated as follows
*DNAm*Age=$F^{-1}$(*b*_0_*+b*_1_*CpG*_1_*+ . . . +b*_p_*CpG*_p_)

where $F^{-1}\left( y \right)$ denotes the mathematical inverse of the function F(.). Thus, the regression model can be used to predict to transformed age value by simply plugging the beta values of the selected CpGs into the formula.

### **Defining Properties of the log linear transformation**

As indicated by its name, the “log-linear” function, has a logarithmic dependence on age before the average age of sexual maturity (of the species) and a linear dependence after Age at Sexual Maturity (of the species). For the human-marmoset clocks we used the following averages at sexual maturity (in units of years): 13.5 years for humans and 1.1767 years for marmosets.

### Construction

We used a piecewise transformation, parameterized by Age of Sexual Maturity ($A$).

The transformation is F(x), given by

$$F\left( x \right)=g\left( \frac{x+1.5}{A+1.5} \right)\text{ where }g\left( t \right)= \left\{ \begin{aligned} \begin{aligned} \begin{aligned} \log\left( t \right), for 0\leq t\leq1 \\ t-1, for 1\leq t \end{aligned} \end{aligned} \end{aligned} \right.$$

Explicitly, F(x) is given by

$$F\left( x \right)=\left\{ \begin{aligned} \begin{aligned} \begin{aligned} \log\left( \frac{x+1.5}{A+1.5} \right), for 0\leq x\leq A \\ \frac{x-A}{A+1.5}, for A\leq x \end{aligned} \end{aligned} \end{aligned} \right.$$

In order to use this transformation to predict Age on *new samples*, one needs to use the *inverse* transformation, F^-1^(y), given by

$$F^{-1}\left( y \right)= \left\{ \begin{aligned} \begin{aligned} \begin{aligned} \left( A+1.5 \right)*\text{exp}\left( y \right)-1.5, for y\leq0 \\ (A+1.5)y+A, for y\geq0 \end{aligned} \end{aligned} \end{aligned} \right.$$

For predicting age, apply the inverse transformation to coefficient-weighted sum. That is,

$$DNAmAge=F^{-1}\left( x*\beta\right)$$

where $\beta$ is the vector of coefficients and $x$ is the vector of methylation values, with an intercept term.

## The DNAm Age estimate is estimated in two steps.

First, one forms a weighted linear combination of the CpGs whose details can be found in Table

The table reports the probe identifier (cg number) used in the custom Infinium array (HorvathMammalMethylChip40) . The weights used in this linear combination are specified in the respective column entitled "Coef.".

The formula assumes that the DNA methylation data measure "beta" values but the formula could be adapted to other ways of generating DNA methylation data.

1 Horvath, S. DNA methylation age of human tissues and cell types. *Genome Biol* **14**, R115, doi:10.1186/gb-2013-14-10-r115 (2013).
